# Neuroprotective Effects of Vanillyl Alcohol in *Gastrodia elata* Blume Through Suppression of Oxidative Stress and Anti-Apoptotic Activity in Toxin-Induced Dopaminergic MN9D Cells

**DOI:** 10.3390/molecules16075349

**Published:** 2011-06-24

**Authors:** In Su Kim, Dong-Kug Choi, Hun Jong Jung

**Affiliations:** 1 Department of Biotechnology, Konkuk University, Chungju 380-701, Korea; 2 Department of Occupation and Environment, University of Konkuk Postgraduate Medical, Chungju 380-701, Korea

**Keywords:** vanillyl alcohol, MPP^+^, MN9D cells, reactive oxygen species, neuroprotection

## Abstract

*Gastrodia elata* Blume (GE) has long been used in oriental countries as a traditional herbal medicine to relieve symptoms associated with neurological ailments such as vertigo, general paralysis and epilepsy. In this study, we have investigated the effects of GE extracts and its major bioactive components on 1-methyl-4-phenylpyridinium (MPP^+^)-treated MN9D dopaminergic cells, a classic *in vitro* model for Parkinson’s disease (PD). We found that vanillyl alcohol effectively inhibited the cytotoxicity and improved cell viability in MPP^+^-induced MN9D dopaminergic cells. The underlying mechanisms of vanillyl alcohol action were also studied. Vanillyl alcohol attenuated the elevation of reactive oxygen species (ROS) levels, decreased in the Bax/Bcl-2 ratio and poly (ADP-ribose) polymerase proteolysis. These results indicate that vanillyl alcohol protected dopaminergic MN9D cells against MPP^+^-induced apoptosis by relieving oxidative stress and modulating the apoptotic process and is therefore a potential candidate for treatment of neurodegenerative diseases such as Parkinson’s disease.

## 1. Introduction

Parkinson’s disease (PD) is one of the most common neurodegenerative disorders. Approximately 2% of the population over 65 years of age is affected by this disease, although incidence varies across age, gender, and race [1,2,3]. The pathological hallmark of PD is a loss of dopaminergic neurons in the substantia nigra pars compacta (SNpc) and a subsequent loss of dopamine in the striatum. Accumulating evidence supports the notion that oxidative stress and activation of the apoptotic cascade play a pivotal role in the neurodegeneration associated with PD [4,5,6], therefore, the suppression of dopaminergic neuronal cell death by regulation of intracellular reactive oxygen species (ROS) and modification of the apoptotic cascade may have therapeutic benefits, leading to alleviation of the progression of neurodegeneration. Insights into PD pathogenesis have been achieved experimentally using the neurotoxin 1-methyl-4-phenylpyridinium (MPP^+^), which is the active metabolite of 1-methyl-4-phenyl-2,3,6-tetrahydropyridine (MPTP). 1-Methyl-4-phenylpyridinium (MPP^+^) has been shown to selectively and potently inhibit complex I of the mitochondrial electron transport chain, and it induces a syndrome closely resembling PD in cellular and animal models [7,8].

*Gastrodia elata* Blume (GE) has been used in Asia since ancient times as a traditional herbal medicine with numerous therapeutic applications. GE is effectively used as an anticonvulsant, analgesic and sedative in vertigo, general paralysis, epilepsy and tetanus [9]. GE extracts have a neuronal protective action due to the presence of amyloid β-peptide, and the protection was associated with increased dopamine concentration and decreased dopamine turnover in striatum [10]. GE also inhibits glutamate-induced apoptosis in neurons [11], inhibits neuronal damage in kainite-induced seizure [12], inhibits MPP^+^-induced cytotoxicity in SH-SY5Y cells [13], and protects against neuronal cell damage after transient global brain ischemia [14]. Its major components are vanillyl alcohol, phenolic compounds, organic acids, glucose, beta-sitosterol, hydroxybenzyl alcohol, vanillin, gastrodin and hydroxybenzaldehyde [15,16].

The purpose of this study was to investigate the effects of GE extract and its major bioactive compounds on MPP^+^-induced neurotoxicity in dopaminergic MN9D cells. We examined the neuroprotective effect of GE extract and vanillyl alcohol on neuronal cell viability, expression of Bcl-2 and Bax, and poly (ADP-ribose) polymerase (PARP) proteolysis in MPP^+^-induced MN9D neurotoxicity, in order to provide possible therapeutic applications for prevention and treatment of PD.

## 2. Results and Discussion

### 2.1. Effect of GE on Cell Viability in MN9D Cells

**Figure 1:**
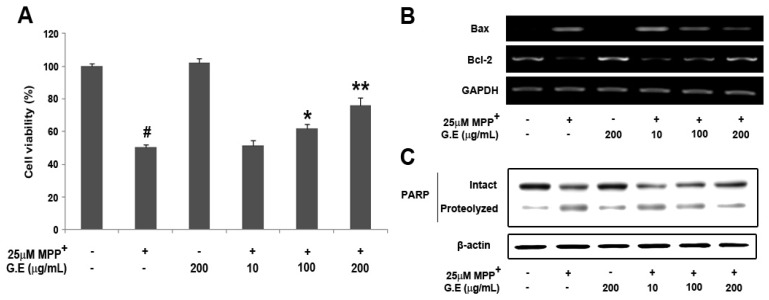
Effect of GE on MPP^+^-induced neuronal cell death in dopaminergic MN9D cells. Cells were exposed to 25 μM MPP^+^ in the absence or presence of GE (10, 100, 200 µg/mL), and cell viability was assessed by MTT assay (**A**); The mRNA expression of Bax and Bcl-2 was determined by RT-PCR (**B**); and PARP proteolysis was analyzed by immunoblot analysis (**C**). Data are expressed as the percentage of values in untreated control cultures and are means ± S.E.M. of three independent experiments in triplicate. ^#^ *P* < 0.05, compared with the control group. * *P* < 0.05, compared with the MPP^+^-treated group (one-way ANOVA followed by Bonferroni *post hoc* test).

The effect of GE on MPP^+^-induced MN9D cells was evaluated using an established MTT assay. Treatment with GE alone did not cause any cytotoxic effects on cell viability up to the highest concentration tested (200 µg/mL), however, the cell viability of MN9D cells exposed to 25 μM MPP^+^ for 48 h decreased significantly to 50.5 ± 1.4%. Pretreatment with GE extract (10, 100, 200 µg/mL) for 4 h prior to the addition of MPP^+^ rescued the MPP^+^-induced decrease in viability by 51.7 ± 3.2%, 62.07 ± 2.3% and 76.3 ± 4.6% in a dose-dependent manner (Figure 1A). We further investigated whether GE affected Bcl-2 and Bax expression and poly(ADP-ribose) polymerase proteolysis in the MPP^+^-induced MN9D cells. The present study demonstrated that MPP^+^-treatment significantly increased Bax expression and PARP cleavage, but significantly decreased Bcl-2 expression levels. GE effectively inhibited both the increase in Bax/Bcl-2 ratio and PARP proteolysis in a dose-dependent manner (Figures 1B and 1C). Taken together, the results suggest that GE might have a protective effect against MPP^+^-induced cytotoxicity in MN9D cells. Previous studies showed that GE extract has a protective effect against neuronal cell damage, as well as free radical scavenging and antioxidant effects [11,17,18]. The protective effect of GE was also confirmed in neuroblastoma SH-SY5Y cells [13].

### 2.2. Effect of Vanillyl Alcohol on Cell Viability in MN9D Cells

**Figure 2:**
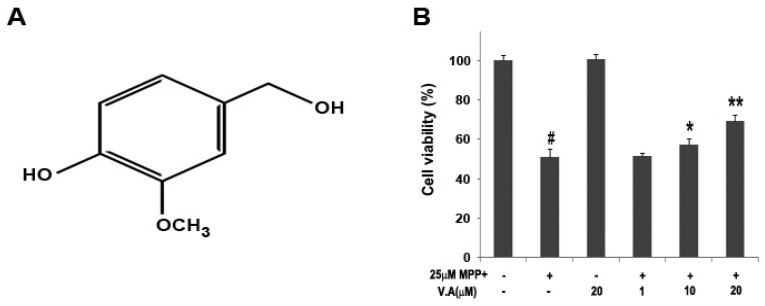
The chemical structure of vanillyl alcohol (**A**); Effect of vanillyl alcohol on cell viability in MN9D cells exposed to MPP^+^ (**B**). Cells were exposed to 25 μM MPP^+^ in the absence or presence of vanillyl alcohol (1, 10, 20 µM) and cell viability was assessed by MTT assay. Data are expressed as the percentage of values in untreated control cultures and are means ± S.E.M. of three independent experiments in triplicate. ^#^ *P* < 0.05, compared with the control group. ^*^ *P* < 0.05, compared with the MPP^+^-treated group (one-way ANOVA followed by Bonferroni *post hoc* test).

The major compounds in GE are vanillyl alcohol, 4-hydroxybenzylaldehyde, vanillin and gastrodin, and these compounds are known to work in the brain after passing through the blood brain barrier [19,20] and display various biological activities such as anti-oxidant, anti-asthmatic, antimicrobial, and antimutagenic effects [19,21,22]. Among those we examined, vanillyl alcohol effectively inhibited the loss of cell viability in a dose-dependent manner according to the 3-(4,5-dimethylthiazol-2-yl)-2,5-diphenyltetrazolium bromide (MTT) assay in MN9D dopaminergic cells. The viability of cells was increased to 51.5 ± 1.4%, 57.5 ± 2.8%, and 69.1 ± 3.1% when cells were pretreated with vanillyl alcohol at 1, 10, and 20 µM, respectively (Figure 2B).

**Figure 3:**
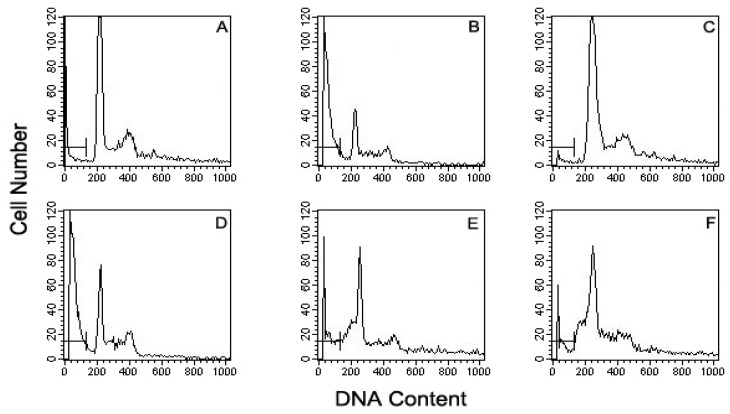
Effect of vanillyl alcohol against MPP^+^-induced neurotoxicity in MN9D cells by flow cytometric DNA analysis. (**A**). Control cells; (**B**). the cells exposed to 25 μM MPP+ alone; (**C**). the cells exposed to 20 μM alone; (**D–F**). the cells pre-treated with vanillyl alcohol at 1, 10, 20 μM, respectively, in the presence of 25 μM MPP^+^. Bar (├─┤) represents a sub-G0/G1 or hypodiploid DNA fraction.

To further verify the inhibitory effect of vanillyl alcohol on MPP^+^-induced MN9D cells, the cells were labeled with propidium iodide and quantitatively analyzed by flow cytometry. When the cells were incubated in medium alone, a typical single peak of nuclei with diploid DNA content was observed (Figure 3A) and there was only about 2%~3% cell death. In the presence of 25 µM MPP^+^, a characteristic hypodiploid DNA content peak indicative of sub-G0-G1 apoptotic populations was distinguishable. Treatment with 25 µM MPP^+^ resulted in apoptotic population of 37.6 ± 1.2% (Figure 3B). Following treatment with vanillyl alcohol (1, 10 and 20 µM), the proportion of apoptotic cells was reduced to 35.4 ± 1.9%, 27.6 ± 2.1%, and 16.8 ± 2.8%, respectively, in a concentration-dependent manner (Figures 3D–3F).

### 2.3. Free Radical Scavenging Activities of Vanillyl Alcohol by ESR

**Figure 4:**
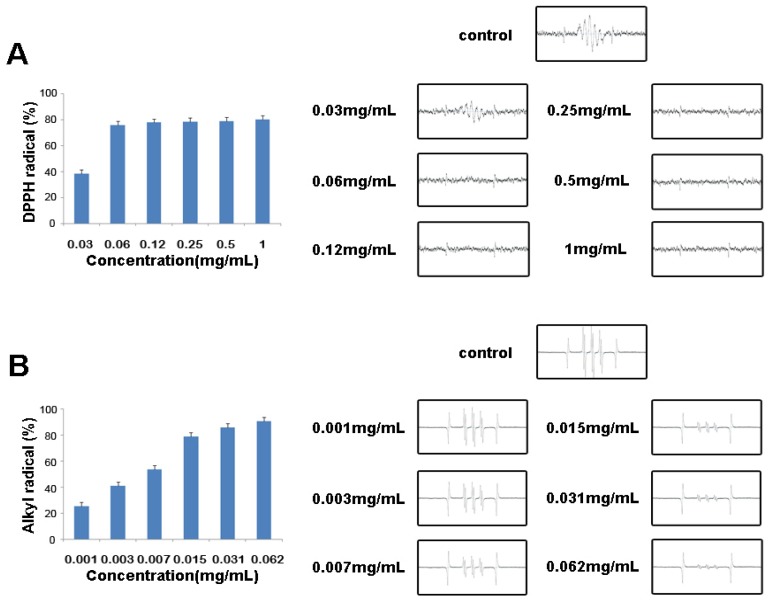
Effect of vanillyl alcohol on the free radical scavenging activity. (**A**). Left: relationship between the signal intensity of DPPH radical and the various concentrations of vanillyl alcohol. Right: ESR spectra of DPPH radicals recorded; (**B**). Left: relationship between the signal intensity of the POBN-alkyl radicals and the various concentrations of vanillyl alcohol. Right: ESR spectra of 2-(4-pyridyl-1-oxide)-*N*-*t*-butylnitrone (POBN)-trapped alkyl radicals recorded.

The free radical scavenging activities of vanillyl alcohol were examined using ESR spectroscopy. DPPH is a stable free radical and accepts an electron or hydrogen radical to produce a stable diamagnetic molecule that can be used to evaluate the free radical scavenging activity of natural antioxidants. The capacity of vanillyl alcohol to scavenge DPPH was measured by ESR spectrometry, and the results are shown in Figure 4A. The DPPH radical scavenging activities of vanillyl alcohol at various concentrations (0.03, 0.06, 0.12, 0.25, 0.5 and 1 mg/mL) were 38.42, 76.08, 77.95, 78.16, 80.29, and 81.10%, respectively, with an IC_50_ value of 0.04 mg/mL. The alkyl radical spin adduct of 4-POBN/free radicals was generated from AAPH at 37 °C for 30 min, and a decrease of ESR signals was observed with the dose increment of GE (Figure 4B). The alkyl radical scavenging activities of vanillyl alcohol (0.001, 0.003, 0.007, 0.015, 0.031 and 0.062 mg/mL) were 25.66, 41.25, 53.81, 78.88, 83.15, and 90.72%, respectively, with an IC_50_ value of 0.006 mg/mL.

The data suggest that vanillyl alcohol is a powerful antioxidant with radical scavenging activity for DPPH and alkyl radicals, a finding that is consistent with earlier reports that vanillyl alcohol shows significant radical scavenging activity for DPPH [23,24]. Several studies also reported that vanillyl alcohol shows antioxidant effect in epileptic seizures in rats [25] and reduces lipid peroxidation in Gerbil brain homogenates [26].

**Figure 5:**
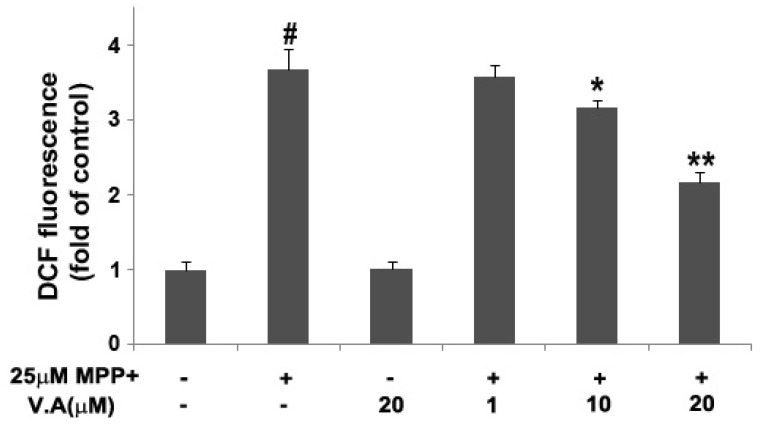
Effects of VA on MPP^+^-induced reactive oxygen species production. Cells were exposed to 25 µM MPP^+^ with or without different concentration of VA (1, 10, 20 µM) for 48 h. ROS generation was detected by fluorometric analysis using DCFH-DA. Data are means ± S.E.M. of three independent experiments in triplicate. ^#^ *P* < 0.05, compared with control group; * *P* < 0.05, ** *P* < 0.01 compared with MPP^+^-treated group in one-way ANOVA followed by Bonferroni *post hoc* test.

Moreover, we measured reactive oxygen species generation in MN9D cells exposed to 25 µM MPP^+^ by fluorometric analysis using DCFH-DA. Cells exposed to MPP^+^ displayed an obvious increase in DCF fluoresence at time- dependent when compared to the control cultures. The DCF fluoresence in cells exposed to 25 µM MPP^+^ at 48 h was 3.67-fold higher than the control group. However, treatment with VA effectively reduced the reactive oxygen species generation and the suppressing effects strengthened with the increase of concentration of VA. The DCF fluoresence in cells incubated with 1, 10 and 20 µM of VA were attenuated to 3.57 ± 0.14, 3.16 ± 0.09, and 2.17 ± 0.13, respectively (Figure 5).

### 2.4. Vanillyl Alcohol Affects the Expression of Bcl-2 and Bax in MPP^+^-Treated Cells

**Figure 6:**
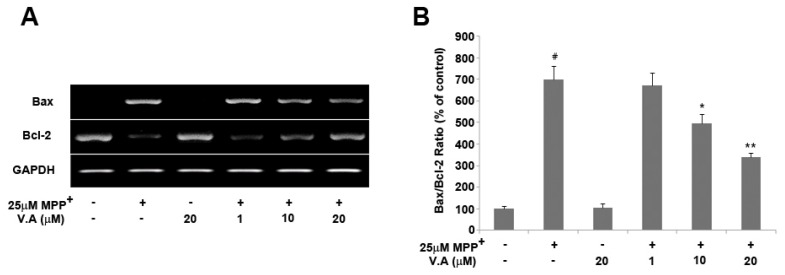
Effects of vanillyl alcohol on the expression of Bcl-2 and Bax in MN9D cells. Cells were treated with 25 μM MPP^+^ in the absence or presence of vanillyl alcohol, and total RNA was collected for expression analysis. The levels of Bax and Bcl-2 were quantitated by densitometric analysis (**A**) and the Bax/Bcl-2 ratio was determined (**B**). Data are means ± S.E.M. of three independent experiments in triplicate. ^#^ *P* < 0.05, compared with control group. * *P* < 0.05, ** *P* < 0.01, compared with MPP^+^-treated group (one-way ANOVA followed by Bonferroni *post hoc* test).

Bcl-2 family proteins are key regulators of apoptosis and include both anti-apoptotic and pro-apoptotic proteins [27]. Cell survival in the apoptotic cascade depends mostly on the balance between the pro- and anti-apoptotic proteins of the Bcl-2 family. The Bax/Bcl-2 ratio may better predict the cell decision between survival and death than the absolute concentrations of either Bax or Bcl-2 alone, and any shift in the balance of pro- and anti-apoptotic proteins may affect cell death [27]. In this study, we investigated whether vanillyl alcohol had any effect on the expression of Bcl-2 and Bax in MPP^+^-treated dopaminergic MN9D cells using expression analysis. The present study demonstrated that 25 µM MPP^+^ treatment significantly enhanced the expression of Bax and concomitantly decreased the levels of Bcl-2. However, vanillyl alcohol treatment substantially suppressed Bax mRNA expression, while expression of Bcl-2 was significantly recovered in a dose-dependent manner. The Bax/Bcl-2 ratio in cells exposed to 25 µM MPP^+^ was 7-fold higher than that in the control group, while in cells pre-treated with 1, 10, and 20 µM vanillyl alcohol, the ratio of Bax to Bcl-2 reversed in a dose-dependent fashion (Figure 6). Our results show that vanillyl alcohol treatments significantly reduced the expression of pro-apoptotic Bax and increased the expression of anti-apoptotic Bcl-2 in a dose-dependent manner, suggesting that vanillyl alcohol treatment shifted the balance between pro- and anti-apoptotic members towards cell survival. Vanillyl alcohol treatment alone did not significantly alter the Bax/Bcl-2 ratio.

### 2.5. Vanillyl Alcohol Suppresses MPP^+^-Induced PARP Proteolysis

**Figure 7:**
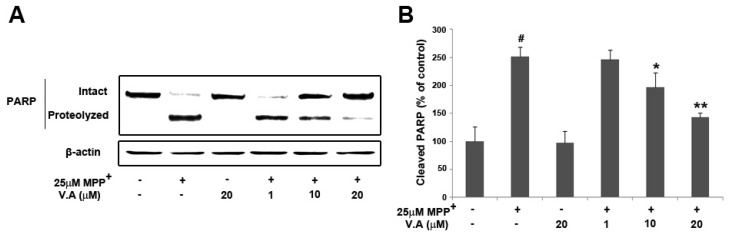
Vanillyl alcohol inhibits MPP+-induced PARP proteolysis. PARP proteolysis was analyzed by immunoblot analysis (**A**) and the levels of cleaved PARP were quantitated by densitometric analysis (**B**). Data are means ± S.E.M. of three independent experiments in triplicate. ^#^ *P* < 0.05, compared with control group; * *P* < 0.05, ** *P* < 0.01 compared with MPP+-treated group in one-way ANOVA followed by Bonferroni *post hoc* test.

The cleavage of PARP is another hallmark of apoptosis [28,29]. Therefore, we further investigated PARP cleavage in MPP^+^-induced MN9D cells. MPP^+^ induces an obvious increase in the cleavage of PARP when compared to control cultures [30]. Cleavage of PARP was detected using polyclonal antibody against cleaved PARP fragments (85 kDa). Following treatment with 25 µM MPP^+^, PARP proteolysis was significantly increased to 251.14 ± 17.39%, while the level of cleaved PARP with vanillyl alcohol treatment (1, 10 and 20 µM) was dose-dependently decreased to 246.28 ± 17.33%, 196.34 ± 26.80%, and 143.24 ± 7.97% (Figure 7). Vanillyl alcohol treatments effectively attenuated MPP^+^-induced PARP cleavage in a dose-dependent manner, indicating that the protective effect of vanillyl alcohol is associated with the inhibition of the downstream apoptotic signaling pathways to prevent the activation of PARP proteolysis. Based on these observations, vanillyl alcohol may modulate the expression of Bcl-2 family proteins in response to MPP^+^ treatment, regulating a succession of mitochondria-mediated downstream molecular events including the activation of PARP.

## 3. Experimental

### 3.1. Materials

1-Methyl-4-phenylpyridinium (MPP^+^), *2*,2-azobis(2-amidinopropane) hydrochloride (AAPH), 1,1-diphenyl-2-picrylhydrazyl (DPPH), (4-pyridyl-1-oxide)-*N*-*tert*-butylnitrone (4-POBN), 3-(3,4-dimethylthiazol-2-yl)-2,5-diphenyltetrazolium bromide (MTT) and vanillyl alcohol were obtained from Sigma Aldrich (St. Louis, MO, USA). Six-well and 96-well tissue culture plates and 100-mm culture dishes were purchased from Nunc Inc. (Naperville, IL, USA). Dulbecco’s modified Eagle’s medium (DMEM) and fetal bovine serum (FBS) were from Gibco-BRL Technologies (Carlsbad, CA, USA). Propidium iodide (PI) was supplied by BD Clontech (Mountain View, CA, USA). The antibodies against PARP and β-actin were obtained from Cell Signaling Co, (Boston, MA, USA). All other chemicals used in this study were analytical grade and were obtained from Sigma Chemical Co. (St. Louis, MO, USA) unless otherwise indicated.

### 3.2. Preparation of GE

*Gastrodia elata* Blume (GE) was purchased in the traditional herb market and was authenticated based on its microscopic and macroscopic characteristics. A voucher specimen (CA04-048) has been deposited at the Plant Extract Bank, Korea. To obtain the ethanol extract, 100 g of GE was added to 95% ethanol and extraction was performed by heating at 50 °C for 7 days; it was then concentrated with a rotary evaporator and lyophilized. The resulting powder (yield, 3.6 g) was dissolved in DMSO and filtered through a 0.22-µM filter before use.

### 3.3. Cell Culture and Treatments

Mouse dopaminergic MN9D cells were kindly provided by Dr. Young J Oh (Yonsei University, Korea). MN9D cells were plated on 96-well plates or 6-well plates coated with 25 µg/mL poly-D-lysine. Cultures were maintained in DMEM supplemented with 10% fetal bovine serum (Invitrogen, San Diego, CA, USA) in an incubator with an atmosphere of 10% CO_2_ at 37 °C for 3 days. Cells were subsequently switched to serum-free N2 medium [31]. All experiments were carried out 24–48 h after cells were seeded. MN9D cells were pretreated with various concentrations (10, 100, 200 µg/mL) of GE and 1, 10, 20 µM vanillyl alcohol for 4 h before incubation in medium containing 25 µM MPP^+^. The control cells were treated with the same medium without drugs.

### 3.4. Assessment of Cell Viability

Cell viability was measured by the quantitative colorimetric MTT assay, showing the mitochondrial activity of living cells as previously described [32]. In brief, MTT dissolved in phosphate-buffered saline was added at the end of incubation to a final concentration of 0.5 mg/mL. After incubation for 4 h at 37 °C in a 5% CO_2_ atmosphere, the supernatants were carefully removed and the formazan crystals formed in the viable cells were measured at 550 nm using a microplate reader (Molecular Devices, Sunnyvale, CA, USA).

### 3.5. Isolation of Total RNA and Expression Analysis for Bax and Bcl-2 mRNA

MN9D cells (1 × 10^6^ cells/well) were cultured in 100-mm plates, and the total RNA was isolated by extraction with TRIzol (Invitrogen, San Diego, CA, USA). For the reverse transcription-polymerase chain reaction (RT-PCR), 2.5 µg of total RNA were reverse transcribed using a First Strand cDNA Synthesis kit (Invitrogen, CA, USA). PCR was performed using the prepared cDNA as a template. The following primers were used for PCR: Bcl-2 sense, 5’-CGCAAGCCGGGAGAACAGGG-3’; Bcl-2 anti-sense, 5’-CTGGCAGCCGTGTCTCGGTG-3’; Bax sense, 5’-GCGAGTGTCTCCGGCGAATT-3’; Bax anti-sense, 5’-GCCCCAGTTGAAGTTGCCATCAG -3’; GAPDH sense, 5’-GGCTCTCTGCTCCTCCCTGTTCTA-3’; GAPDH anti-sense 5’-TGCCGTTGAACTTGCCGTGGG-3’. PCR products were electrophoresed on a 1% (w/v) agarose gel and stained with ethidium bromide. Relative expression was quantified densitometrically with the Multi Gauge Version 3.1 system (Fujifilm Co., Japan), and calculated using the reference bands of GAPDH.

### 3.6. Immunoblot Analysis for Cleaved PARP

To obtain the total cell lysate, 0.1 mL (or 0.05 mL) of RIPA buffer [1 × PBS, 1% NP-40, 0.5% sodium deoxycholate, 0.1% SDS, with freshly added protease inhibitor cocktail (Calbiochem, CA, USA)] was added to MN9D cells cultured in 100-mm plates. Cells were scraped, incubated for 10 min on ice, and centrifuged at 14,000 × rpm for 10 min at 4 °C. Protein concentration was determined by the DC protein assay from Bio-Rad (Hercules, CA, USA), and 15 µg of whole cell lysate were loaded for 10% SDS-PAGE. Electrophoresis was performed and the proteins were transferred to PVDF membranes (Millipore, MA, USA) using an electroblotting apparatus (Bio-Rad, CA, USA). The membranes were blocked for 1 h in TBS containing 0.1% Tween-20 and 5% dry milk, and were then incubated overnight with primary antibodies [anti-PARP 1:1000 (Cell Signaling, MA, USA)] followed by incubation for 1 h with horseradish peroxidase-conjugated secondary antibodies (1:10,000) (Santa Cruz, CA, USA). The optical densities of the antibody-specific bands were analyzed by a Luminescent Image Analyzer, LAS-3000 (Fuji, Japan). Data were normalized to the reference bands of β-actin.

### 3.7. Flow Cytometric Detection of Apoptotic Cells

MN9D cells were collected by centrifugation and washed with ice-cold PBS. Pellets were resuspended in ice-cold 70% ethanol and fixed at 4 °C for 24–48 h, washed and resuspended in 1 mL of DNA staining reagent containing 50 µg/mL RNase, 0.1% Triton X-100, 0.1 mM EDTA (pH 7.4), and 50 µg/mL PI. Staining was stable at 4 °C for 30 min [33]. Red fluorescence (DNA) was detected through a 563–607 nm band pass filter by using a FACS Caliber flow cytometer (Becton Dickinson, San Jose, CA, USA.). Ten thousand cells in each sample were analyzed and the percentage of apoptotic cells accumulating in the sub-G1 peak was calculated by Cell Quest software.

### 3.8. Measurement of Free Radical Scavenging Activity

Free radical scavenging activity was evaluated using an electron spin resonance (ESR) spectrometer (JEOL, Tokyo, Japan). DPPH radical scavenging activity was measured as described by Nanjo *et al.* [34]. A sample solution of vanillyl alcohol was added to 60 µM DPPH in methanol solution was incubated for 2 min. Alkyl radicals were generated by AAPH. The reaction mixture containing 10 mM AAPH, 10 mM 4-POBN and vanillyl alcohol with various concentrations in PBS (pH 7.4) were incubated at 37 °C in a water bath for 30 min. The ESR spectrum for each radical was recorded using an ESR spectrometer.

### 3.9. Measurement of Intracellular Reactive Oxygen Species (ROS)

The intracellular ROS production was measured using a non-fluorescent compound 2’,7’-dichlorofluorescein diacetate (DCFH-DA) as previously described [35]. It measures the formation of hydrogen peroxide generated by an oxidative metabolic burst. Viable cells can deacetylate DCFH-DA to 2’,7’-dichlorofluorescin (DCFH), which is not fluorescent. This compound reacts quantitatively with oxygen species within the cell to produce a fluorescent dye 2’,7’-dichlorofluorescein (DCF), which remains trapped within the cell and can be measured to provide an index of ROS level. After the drug treatment, cultures were washed with PBS, loaded with 20 µM DCF-DA for 30min at 37 °C, and then washed again with PBS. DCF fluorescence was analyzed using a fluorescence plate reader (Spectramax M2e, Molecular Devices) at excitation and emission wavelengths of 490 and 530 nm.

### 3.10. Statistical Analysis

The results were expressed as mean ± S.E.M. of at least three independent experiments performed in triplicate. Statistical analysis was performed by one-way analysis of variance (ANOVA) followed by *post hoc* multiple comparisons using the Bonferroni method in the Sigma Stat 3.1 software (Systat Software Inc., San Jose, CA, USA). A *P* value less than 0.05 was considered statistically significant.

## 4. Conclusions

Here we have demonstrated that vanillyl alcohol has neuroprotective effects against MPP^+^-induced cytotoxicity in dopaminergic MN9D cells by multiple lines of evidence. The data revealed that vanillyl alcohol protected cells by reducing MPP^+^-induced cell loss, decreased the number of apoptotic cells and PARP proteolysis, and decreased the Bax/Bcl-2 ratio. Vanillyl alcohol-mediated neuroprotection is due, in part, to inhibition of the mitochondrial apoptotic pathway. The anti-oxidative and anti-apoptotic properties of vanillyl alcohol might play a major role in rendering a protective action against MPP^+^-induced cytotoxicity in dopaminergic MN9D cells. This study reports for the first time that vanillyl alcohol could ameliorate MPP^+^-induced oxidative stress and apoptosis in dopaminergic MN9D cells and exert neuroprotective activity, which may be partly responsible for the neuroprotective efficacies of GE. However, further studies on the detailed mechanism and in animal models of PD and comparison with known anti-parkinsonian agents should be conducted.

## References

[1] Dick S., Semple S., Dick F., Seaton A. (2007). Occupational titles as risk factors for Parkinson’s disease. Occup. Med. (Lond).

[2] Dorsey E.R., Constantinescu R., Thompson J.P., Biglan K.M., Holloway R.G., Kieburtz K., Marshall F.J., Ravina B.M., Schifitto G., Siderowf A. (2007). Projected number of people with Parkinson disease in the most populous nations, 2005 through 2030. Neurology.

[3] Elbaz A., Bower J.H., Maraganore D.M., McDonnell S.K., Peterson B.J., Ahlskog J.E., Schaid D.J., Rocca W.A. (2002). Risk tables for parkinsonism and Parkinson’s disease. J. Clin. Epidemiol..

[4] Mattson M.P. (2000). Apoptosis in neurodegenerative disorders. Nat. Rev. Mol. Cell Biol..

[5] Pieczenik S.R., Neustadt J. (2007). Mitochondrial dysfunction and molecular pathways of disease. Exp. Mol. Pathol..

[6] Szeto H.H. (2006). Mitochondria-targeted peptide antioxidants: Novel neuroprotective agents. AAPS J..

[7] Eberhardt O., Schulz J.B. (2003). Apoptotic mechanisms and antiapoptotic therapy in the MPTP model of Parkinson’s disease. Toxicol. Lett..

[8] Przedborski S., Jackson-Lewis V. (1998). Mechanisms of MPTP toxicity. Mov. Disord..

[9] Ojemann L.M., Nelson W.L., Shin D.S., Rowe A.O., Buchanan R.A. (2006). Tian ma, an ancient Chinese herb, offers new options for the treatment of epilepsy and other conditions. Epilepsy Behav..

[10] Shuchang H., Qiao N., Piye N., Mingwei H., Xiaoshu S., Feng S., Sheng W., Opler M. (2008). Protective effects of gastrodia elata on aluminium-chloride-induced learning impairments and alterations of amino acid neurotransmitter release in adult rats. Restor. Neurol. Neurosci..

[11] Lee Y.S., Ha J.H., Yong C.S., Lee D.U., Huh K., Kang Y.S., Lee S.H., Jung M.W., Kim J.A. (1999). Inhibitory effects of constituents of Gastrodia elata Bl. on glutamate-induced apoptosis in IMR-32 human neuroblastoma cells. Arch. Pharm. Res..

[12] Kim H.J., Moon K.D., Oh S.Y., Kim S.P., Lee S.R. (2001). Ether fraction of methanol extracts of Gastrodia elata, a traditional medicinal herb, protects against kainic acid-induced neuronal damage in the mouse hippocampus. Neurosci. Lett..

[13] An H., Kim I.S., Koppula S., Kim B.W., Park P.J., Lim B.O., Choi W.S., Lee K.H., Choi D.K. (2010). Protective effects of Gastrodia elata Blume on MPP+-induced cytotoxicity in human dopaminergic SH-SY5Y cells. J. Ethnopharmacol..

[14] Kim H.J., Lee S.R., Moon K.D. (2003). Ether fraction of methanol extracts of Gastrodia elata, medicinal herb protects against neuronal cell damage after transient global ischemia in gerbils. Phytother. Res..

[15] Baek N.I., Choi S.Y., Park J.K., Cho S.W., Ahn E.M., Jeon S.G., Lee B.R., Bahn J.H., Kim Y.K., Shon I.H. (1999). Isolation and identification of succinic semialdehyde dehydrogenase inhibitory compound from the rhizome of Gastrodia elata Blume. Arch. Pharm. Res..

[16] Wu C.R., Hsieh M.T., Huang S.C., Peng W.H., Chang Y.S., Chen C.F. (1996). Effects of Gastrodia elata and its active constituents on scopolamine-induced amnesia in rats. Planta Med..

[17] Ha J.H., Lee D.U., Lee J.T., Kim J.S., Yong C.S., Kim J.A., Ha J.S., Huh K. (2000). 4-Hydroxy- benzaldehyde from Gastrodia elata B1. is active in the antioxidation and GABAergic neuromodulation of the rat brain. J. Ethnopharmacol..

[18] Liu J., Mori A. (1992). Antioxidant and free radical scavenging activities of Gastrodia elata Bl. and Uncaria rhynchophylla (Miq.) Jacks. Neuropharmacology.

[19] Jang Y.W., Lee J.Y., Kim C.J. (2010). Anti-asthmatic activity of phenolic compounds from the roots of Gastrodia elata Bl. Int. Immunopharmacol..

[20] Lin L.C., Chen Y.F., Lee W.C., Wu Y.T., Tsai T.H. (2008). Pharmacokinetics of gastrodin and its metabolite p-hydroxybenzyl alcohol in rat blood, brain and bile by microdialysis coupled to LC-MS/MS. J. Pharm. Biomed. Anal..

[21] An S.J., Park S.K., Hwang I.K., Choi S.Y., Kim S.K., Kwon O.S., Jung S.J., Baek N.I., Lee H.Y., Won M.H. (2003). Gastrodin decreases immunoreactivities of gamma-aminobutyric acid shunt enzymes in the hippocampus of seizure-sensitive gerbils. J. Neurosci. Res..

[22] Cerrutti P., Alzamora S.M. (1996). Inhibitory effects of vanillin on some food spoilage yeasts in laboratory media and fruit purees. Int. J. Food Microbiol..

[23] Reddy K.K., Ravinder T., Prasad R.B., Kanjilal S. (2011). Evaluation of the antioxidant activity of capsiate analogues in polar, nonpolar, and micellar media. J. Agric. Food Chem..

[24] Shyamala B.N., Naidu M.M., Sulochanamma G., Srinivas P. (2007). Studies on the antioxidant activities of natural vanilla extract and its constituent compounds through *in vitro* models. J. Agric. Food Chem..

[25] Hsieh C.L., Tang N.Y., Chiang S.Y., Hsieh C.T., Lin J.G. (1999). Anticonvulsive and free radical scavenging actions of two herbs, Uncaria rhynchophylla (MIQ) Jack and Gastrodia elata Bl., in kainic acid-treated rats. Life Sci..

[26] Jung T.Y., Suh S.I., Lee H., Kim I.S., Kim H.J., Yoo H.S., Lee S.R. (2007). Protective effects of several components of Gastrodia elata on lipid peroxidation in gerbil brain homogenates. Phytother. Res..

[27] Cory S., Adams J.M. (2002). The Bcl2 family: Regulators of the cellular life-or-death switch. Nat. Rev. Cancer.

[28] Lazebnik Y.A., Kaufmann S.H., Desnoyers S., Poirier G.G., Earnshaw W.C. (1994). Cleavage of poly(ADP-ribose) polymerase by a proteinase with properties like ICE. Nature.

[29] Le D.A., Wu Y., Huang Z., Matsushita K., Plesnila N., Augustinack J.C., Hyman B.T., Yuan J., Kuida K., Flavell R.A. (2002). Caspase activation and neuroprotection in caspase-3- deficient mice after *in vivo* cerebral ischemia and *in vitro* oxygen glucose deprivation. Proc. Natl. Acad. Sci. USA.

[30] Kitamura Y., Kosaka T., Kakimura J.I., Matsuoka Y., Kohno Y., Nomura Y., Taniguchi T. (1998). Protective effects of the antiparkinsonian drugs talipexole and pramipexole against 1-methyl-4-phenylpyridinium-induced apoptotic death in human neuroblastoma SH-SY5Y cells. Mol. Pharmacol..

[31] Bottenstein J.E., Sato G.H. (1979). Growth of a rat neuroblastoma cell line in serum-free supplemented medium. Proc. Natl. Acad. Sci. USA.

[32] Datki Z., Juhasz A., Galfi M., Soos K., Papp R., Zadori D., Penke B. (2003). Method for measuring neurotoxicity of aggregating polypeptides with the MTT assay on differentiated neuroblastoma cells. Brain Res. Bull..

[33] Telford W.G., King L.E., Fraker P.J. (1991). Evaluation of glucocorticoid-induced DNA fragmentation in mouse thymocytes by flow cytometry. Cell Prolif..

[34] Nanjo F., Goto K., Seto R., Suzuki M., Sakai M., Hara Y. (1996). Scavenging effects of tea catechins and their derivatives on 1,1-diphenyl-2-picrylhydrazyl radical. Free Radic. Biol. Med..

[35] Bass D.A., Parce J.W., Dechatelet L.R., Szejda P., Seeds M.C., Thomas M. (1983). Flow cytometric studies of oxidative product formation by neutrophils: A graded response to membrane stimulation. J. Immunol..

